# Classic biomarkers in exercise research for brain health promotion: From cells to communities

**DOI:** 10.1002/alz.088039

**Published:** 2025-01-09

**Authors:** Cindy K Barha

**Affiliations:** ^1^ Libin Cardiovascular Institute, Calgary, AB, Canada; Hotchkiss Brain Institute, Calgary, AB, Canada; University of Calgary, Calgary, AB Canada

## Abstract

There is growing recognition that exercise is a potent stimulus for improving cognitive and brain function in both humans and rodents. While the mechanisms underlying the effect of exercise on cognition are very likely multifactorial, it is clear that the secretion and function of several classical factors are involved. Work done in rodents implicates several growth factors, including BDNF, IGF‐1, and VEGF, in the ability of exercise to induce neuroplastic changes within key brain regions associated with learning and memory. However, confirming these growth factors as mediators of exercise efficacy in human trials has been less successful. This is likely related to several things, including a disconnect between human and rodent exercise studies in terms of design and analysis. We suggest that biological sex alone and in interaction with important genetic polymorphisms implicated in cognitive decline as vital moderating factors that must be considered in the search for exercise biomarkers.

A sex difference exists in exercise efficacy for cognition, with older females showing greater benefits for executive functions than older males. The ε4 variant of the apolipoprotein E (*APOE*) gene moderates the effect of exercise on cognition; although the direction of the relationship is unclear. Biological sex may interact with the *APOE* gene to further moderate exercise effects on cognition. Our work indicates that biological sex interacts with APOE4 status to moderate exercise effects on the brain in both humans and rodents. Additionally, the BDNFval66met polymorphism also influences exercise efficacy for promoting cognition and neuroplasticity in a sex‐dependent manner. How these polymorphisms impact classic exercise biomarkers in males and females are important avenues of research. Work in rodents indicates that systemic/central sex hormones are further involved in the effect of exercise; though which hormones and to what extent is still unclear. There is an overall paucity of studies in humans that have investigated the role of different sex hormones in mediating the cognitive‐response to exercise in the context of aging. A greater appreciation of tissue‐specific effects of exercise on sex hormone production and growth factors and the synergistic interaction between biological sex and genetics, will help move this field forward.

